# The Book World of Medicine and Science

**Published:** 1911-10-21

**Authors:** 


### The Book World of Medicine and Science.

MANCHESTER AND SALFORD SANITARY ASSOCIATION: Proceedings at a Conference on the Care of the Feeble-minded held at Manchester on Thursday, May 4, 1911. (Manchester: Sherratt and Hughes. 1911. Price 6d.)

THIS report is a useful contribution to the literature of the subject in that it sets out views from different standpoints, those of physicians, educationists, public health officers, civic authorities, and teachers. As might be expected, such opinions are often at variance, but this is hardly to be wondered at, seeing the complexity of the subject.

BIOGRAPHICAL REMINISCENCES: A MEMOIR. By Sir SAMUEL WILKS, Bart., M.D., LL.D., F.R.S. (London: Adlard and Son, Bartholomew Close, E.C. 1911. Price 3s. 6d. net.)

THE regret that the reader feels on laying down this book is that Sir Samuel has been so parsimonious in his recollections. To the modern generation of medical men, the memoirs of one who is regarded, and justly so, as the doyen and G.O.M. of the profession in this country, are brimful of interest. It is the personal that appeals in such books, and although Sir Samuel is autobiographical in part he gives us just sufficient to whet our appetite for more. Let us hope that he will see his way clear to give us more before very long. As indemnification against the loss of the personal autobiographical element, Sir Samuel gives us an interesting and most instructive résumé of the work done at Guy's during his time, mainly in the shape of a synopsis of the Reports. To most of us this summary will appeal strongly, as it is an endeavour to bring into focus the rather inchoate elements which are scattered through the voluminous literature which has accumulated during the last fifty years. To some for example it will be astonishing to know that at the commencement of that period no autopsy record of a case of syphilis had been published; Wilks was the first to point out the importance of the visceral lesions in that disease. Similarly the notes on Bright's disease, pyæmia, pernicious anæmia, and typhoid fever are valuable contributions to the history of English medicine. The little volume is very readable and from start to finish replete with interest, so that it should appeal not only to those who admire the author and his work but to every medical man.

THE SURGEON'S LOG: BEING IMPRESSIONS OF THE FAR EAST. By J. JOHNSTON ABRAHAM. Illustrated. (London: Chapman and Hall, Ltd. 1911. Price 7s. 6d.)

MANY young doctors go for a voyage, but there are few who can enter into the spirit of a seafaring life with such obvious success as Dr. Johnston Abraham, and fewer still who could commit to paper so delightfully the kaleidoscopic impressions encountered on a voyage to the East. As a mere descriptive traveller's tale this book will hold its own, but with the added interest of the personalities so vividly presented by this "ship's doc." the book becomes fascinating. We are quite sure that Dr. Abraham will have to be held responsible for many a decision to "do a voyage" which will be taken by the coming generation of medical men. But of his own particular duties and medical work the author makes very modest mention, probably from lack of material, as his ship carried no ordinary passengers, so the lay reader need have no fear of coming across any boring technical matter. The numerous photographs scattered throughout the book are not only successful reproductions but are also an additional pleasure, depicting as they do scenes and incidents in keeping with the characteristic of the book. This is no stilted descriptive guide-book, but one that breathes reality and the joy of living from cover to cover. Others have known what it is to feel the influence of the East, and they will have every sympathy with the author's sentiments in the last chapter; but he does not indulge in much Kiplingesque rhapsody, but narrates everyday trifles and paints the picturesque normalities of the East with a facility and charm worthy to rank high in the world of literature. No other book on a voyage to the Far East has so appealed to us, both in its substance and its setting, as this delightful, easy, and brisk traveller's tale, with its acute portraits and telling descriptions. We feel as if the profession owes Dr. Johnston Abraham a debt of gratitude for his decision to remain in it, and his courage in pronouncing an irrevocable "Sayonara" to the fascination of Eastern voyages.

THE HUNTERIAN LECTURES ON COLOUR-VISION AND COLOUR-BLINDNESS. By Professor F. W. EDRIDGE-GREEN, M.D., F.R.C.S. (London: Kegan Paul, Trench and Co. 1911. Price 3s. 6d. net.)

THE lectures delivered by Professor Edridge-Green before the Royal College of Surgeons last February are now available in book form, and should prove acceptable to many who are interested in the subject of vision and colour-blindness. In a short preface a few details are given of the structure of the eye, for the benefit of others than medical examiners. The second lecture deals with the detection of colour-blindness practically, and gives full particulars of the methods employed and a description of the special lantern used by the author, who at the same time points out the deficiencies of certain other tests in vogue.

THEORY AND PRACTICE OF THYROID THERAPY: A BOOK FOR GENERAL PRACTITIONERS. By H. EWAN WALLER, M.R.C.S., L.R.C.P. (London: John Bale, Sons, and Danielsson. 1911. Pp. 154. Price 5s. net.)

IT is always interesting and sometimes refreshing to read a book written by a general practitioner, but unfortunately his leisure is limited and the output of this class of literature must necessarily be small. Mr. H. E. Waller has chosen the subject of thyroid medication, a theme which lends itself very thoroughly, in the present state of our knowledge, to speculative theorising and bold deduction. In a somewhat discursive style the author discusses the actions and reactions of the thyroid secretion under a great variety of conditions, illustrated by cases in his own practice. Beginning as an ardent disciple of Dr. Leonard Williams, the author has made good use of his own powers of observation, with the result that the book contains a large number of ingenious suggestions on the therapeutics of thyroid extract. Without going into the merits of his deductions or criticising his logic, we may congratulate him as a general practitioner on the earnestness and clinical acumen he has shown himself capable of. Far from anticipating that he will be reproached with having acted on the plan—"when in doubt, give thyroid," we prefer to believe that the account of his wanderings into this little-known land will prove interesting to others and of assistance to some in enabling them to penetrate still further.

